# Influence of Calcination Temperature on the Structure and Antimicrobial Properties of *Arthrospira platensis*-Mediated Zinc Oxide Nanoparticles

**DOI:** 10.3390/pharmaceutics17111367

**Published:** 2025-10-23

**Authors:** Noor Akhras, Abuzer Çelekli, Hüseyin Bozkurt

**Affiliations:** 1 Department of Biochemistry Science and Technology, Faculty of Arts and Science, Gaziantep University, Gaziantep 27310, Türkiye; 2 Department of Biology, Faculty of Art and Science, Gaziantep University, Gaziantep 27310, Türkiye; celekli.a@gmail.com; 3 Department of Food Engineering, Faculty of Engineering, Gaziantep University, Gaziantep 27310, Türkiye; hbozkurt@gantep.edu.tr

**Keywords:** *Arthrospira platensis*, zinc oxide nanoparticles, calcination temperature, antioxidant activity, antimicrobial properties

## Abstract

**Background/Objectives**: *Arthrospira platensis* (*A. platensis*) is a cyanobacterium rich in bioactive compounds with proven antioxidant, antimicrobial, and stabilizing properties, making it an ideal candidate for the green synthesis of zinc oxide nanoparticles (ZnO NPs). This study aimed to synthesize ZnO NPs using *A. platensis* extract and to evaluate the influence of post-synthesis temperature on their physicochemical and antimicrobial properties. **Methods**: ZnO NPs were synthesized via a co-precipitation method using *A. platensis* extract, followed by post-synthesis treatments at 80 °C and 400 °C. Comprehensive characterization was performed using Ultraviolet–Visible Spectroscopy (UV–Vis), Fourier Transform Infrared Spectroscopy (FT–IR), Field Emission Scanning Electron Microscopy (FE–SEM), and Energy Dispersive X-ray Spectroscopy (EDX) to assess optical, structural, and compositional features. Antioxidant activity (DPPH assay) and antimicrobial properties against *Staphylococcus aureus*, *Escherichia coli*, and Candida albicans were also evaluated. **Results**: FE–SEM analysis confirmed a temperature-dependent effect, with ZnO NPs synthesized at 80 °C appearing as polydispersed, irregular aggregates (45.2 ± 8.6 nm), while calcination at 400 °C yielded compact, angular nanoparticles (37.1 ± 6.3 nm). In contrast, pure ZnO NPs were smaller (26.4 ± 4.1 nm), and *A. platensis* extract alone showed amorphous, irregular structures. FTIR spectra demonstrated the involvement of biomolecules in nanoparticle capping and stabilization, whereas EDX analysis revealed that higher calcination reduced organic residues and increased zinc purity. Antioxidant assays indicated a decrease in phenolic and flavonoid content with increasing temperature, leading to reduced DPPH radical scavenging activity. Antimicrobial evaluation showed superior inhibition zones (17.8–26.0 mm) for *A. platensis*-ZnO NPs compared to the crude extract, with *S. aureus* being most susceptible, particularly to the 400 °C nanoparticles. **Conclusions**: The study demonstrates that *A. platensis* extract provides a sustainable and efficient route for ZnO NP biosynthesis. Calcination temperature significantly affects nanoparticle morphology, biochemical composition, and antimicrobial performance. These findings highlight the potential of *A. platensis*-ZnO NPs as eco-friendly antimicrobial agents for biomedical, pharmaceutical, and food preservation applications.

## 1. Introduction

Bacterial antimicrobial resistance (AMR) is a leading global cause of death nowadays. In 2019, the most comprehensive study reported 4.95 million AMR-associated deaths, including 1.27 million directly caused by resistant infections, with the heaviest burden in low- and middle-income countries. Much of the mortality is nowadays attributable to a narrow set of bacterial pathogens such as *Escherichia coli*, *Staphylococcus aureus*, *Klebsiella pneumoniae*, *Streptococcus pneumoniae*, *Acinetobacter baumannii* and *Pseudomonas aeruginosa* that cause life-threatening lower-respiratory, bloodstream, and intra-abdominal infections. By elevating the risk of therapy-refractory infections, AMR undermines the safety of common surgeries, obstetric procedures, and chemotherapy. Consequently, novel, safe, and scalable antimicrobial strategies are required to complement control and infection prevention [1]. New antibiotics are urgently needed because many first-line drugs are losing their effectiveness, while the available treatments for dangerous Gram-negative bacteria remain very limited and sometimes highly toxic [2]. Only a small number of new antibiotics have reached patients in recent years, and most of them belong to the same existing drug families, such as β-lactams (e.g., ceftazidime and avibactam) or tetracyclines (e.g., eravacycline), rather than representing truly novel antibiotic classes [3]. Development is further slowed by biological challenges including bacterial outer membranes, efflux pumps, and enzymes that inactivate drugs, as well as by low commercial incentives and the high cost and complexity of clinical trials [3].

Alongside conventional drug discovery, nanotechnology-based antimicrobials have gained considerable attention as alternative or adjunct approaches to combat resistant infections. Nanoparticles possess unique physical and chemical properties, including their very small size, large surface area compared to volume, adjustable surface features, and controlled ion release [4]. These properties allow them to interact with bacteria in ways that are different from traditional antibiotics. Among them, zinc oxide nanoparticles (ZnO NPs) are especially promising because they combine broad-spectrum antimicrobial activity with relative biocompatibility and low cost of synthesis. Recent reviews highlight that ZnO NPs are effective against both Gram-positive and Gram-negative bacteria, including multidrug-resistant strains [4,5].

The antimicrobial activity of ZnO NPs is mediated by multiple mechanisms. One important pathway is the generation of reactive oxygen species (ROS) such as superoxide and hydroxyl radicals, which damage bacterial membranes, proteins, and DNA. Another involves direct interaction with the negatively charged bacterial cell wall, leading to membrane disruption and leakage of cellular contents. A further mechanism is the controlled release of Zn^2+^ ions, which interferes with essential enzymatic pathways and metabolic processes. Unlike conventional antibiotics, which usually act on a single target, these multimodal mechanisms reduce the likelihood of bacteria developing resistance [6,7]. Besides their effect on planktonic bacteria, ZnO NPs are also active against bacterial biofilms, which are often tolerant to antibiotics [8,9]. Previous studies have also shown that ZnO NPs can effectively penetrate biofilm matrices, disrupt bacterial quorum sensing, and enhance the efficacy of existing antimicrobial treatments against biofilm-embedded pathogens [8,9]. Furthermore, ZnO NPs can act synergistically with antibiotics, lowering the required dose and restoring the activity of drugs that have lost effectiveness. Such combination strategies are being increasingly explored as a way to extend the lifespan of current antibiotics while introducing nanomaterials as novel therapeutic tools [10].

The importance of this field lies not only in antimicrobial performance but also in practical applicability. ZnO NPs are already used in food packaging, wound dressings, and personal care products for their antimicrobial and UV-shielding properties. The shift toward eco-friendly, green synthesis using biological extracts such as cyanobacteria, adds further value by improving biocompatibility and sustainability, while reducing reliance on hazardous chemicals [10,11]. Together, these advances make nanotechnology and ZnO NPs in particular, one of the most promising new antimicrobial approaches to support the control and prevention of infections.

Cyanobacteria are increasingly recognized as photosynthetic microorganisms with significant biotechnological relevance. Their importance is attributed to their ability to synthesize a wide array of nutraceutical and pharmaceutical compounds. Among them, *A. platensis* (Spirulina) is particularly valuable, as it is one of the few cyanobacteria considered safe for human and animal consumption. Its diverse biochemical composition makes it especially important for the development of products with therapeutic potential [6,10,12]. *A. platensis* is a filamentous cyanobacterium, rich in proteins, essential fatty acids, polysaccharides, pigments like phycocyanin, and a variety of phenolic and flavonoid compounds [13].

These bioactive metabolites are responsible for their hypolipidemic, hypoglycemic antioxidant, anti-inflammatory, antimicrobial and anticancer properties, and they contribute to protective effects against a wide range of human diseases. For example, phycocyanin has demonstrated strong antioxidant and anti-inflammatory activity, while phenolics and flavonoids provide antibacterial and antifungal effects [11,14]. In addition to their therapeutic roles, these compounds facilitate the reduction and stabilization of metal precursors, making *A. platensis* an effective natural agent for the green synthesis of zinc oxide nanoparticles with enhanced biomedical applications and environmental applications [8,12,14].

The development of ZnO NPs via green synthesis has emerged as a more sustainable and biocompatible alternative to conventional physical and chemical methods. In contrast to traditional techniques that often rely on hazardous reagents, high energy input, or complex processing steps, green synthesis utilizes natural biological systems to facilitate nanoparticle formation under controlled, low-temperature, and chemically safe conditions [15]. The phytochemical constituents of *A. platensis* such as polyphenols, proteins, and pigments play a crucial role in reducing zinc ions to ZnO nanostructures while at the same time acting as capping and stabilizing agents [16]. This eco-friendly approach aligns with the principles of green chemistry by minimizing environmental toxicity and promoting energy efficient synthesis. However, despite notable progress in synthesis of biogenic ZnO NPs, the impact of post-synthesis thermal treatments, particularly calcination temperature, on the structural integrity, crystallinity and biological functionality of these nanoparticles remains insufficiently explored [6,7].

Calcination temperature is an important parameter that affects several key characteristics of ZnO NPs, including their crystallinity, particle size, morphology, surface area, and defect structure. These physicochemical traits, in turn, directly influence the nanoparticles’ optical, photocatalytic, and antimicrobial properties [7]. Previous study have demonstrated that increasing calcination temperatures can improve crystallinity and reduce organic residues, but may also lead to particle agglomeration or altered surface reactivity [17]. For example, one investigation reported that ZnO NPs calcined at 400 °C exhibited sharper XRD peaks and smaller crystal sizes compared to those dried at room temperature, leading to enhanced antibacterial activity against *E. coli* and *S. aureus* [18].

Yet, the balance between retaining bioactive functional groups from biological capping agents and achieving desirable structural integrity remains delicate. Low-temperature conditions (such as 80 °C) help preserve organic molecules from *A. platensis* that may contribute to synergistic antimicrobial effects, whereas moderate temperature calcination (for example, 400 °C) enhances crystallinity and reduces impurities. Understanding the different effects of low and moderate temperature treatments is essential for optimizing the structural properties of ZnO NPs for specific applications

Although several studies in the literature have explored the influence of calcination temperature on the physicochemical properties of ZnO NPs synthesized via green methods, including those mediated by *A. platensis*, the specific effects of low-temperature drying (80 °C) and moderate calcination (400 °C) remain largely unexamined. Previous research has predominantly focused on high-temperature treatments (often exceeding 500 °C) to enhance crystallinity, particle uniformity, and functional performance [7]. Additionally, another study examined *A. platensis* based ZnO NPs calcined at 160 °C, 300 °C, and 600 °C, highlighting shifts in antioxidant activity and structural morphology [19]. However, these studies did not assess lower temperature limits such as 80 °C, nor did they systematically compare thermal effects on antimicrobial efficacy relative to raw biomass extracts. In this context, the present study is the first to directly compare *A. platensis*-mediated ZnO NPs treated at 80 °C and 400 °C, evaluating their structural characteristics alongside their antibacterial performance against key pathogens. By including comparisons with the raw *A. platensis* extract and pure ZnO NPs, this research offers novel insight into the relation between thermal processing, nanoparticle properties, and antimicrobial functionality thus filling a critical gap in the current understanding of temperature-controlled green nanomaterials.

## 2. Material and Methods

### 2.1. Materials

In this study, a range of analytical grade chemicals and biological materials were used. Standard phenolic and flavonoid compounds including apigenin (C_15_H_10_O_5_, 98%), galangin (C_15_H_10_O_5_, ≥98%), quercetin (C_15_H_10_O_7_, 97%), chlorogenic acid (C_16_H_18_O_9_, 98%), kaempferol (C_15_H_10_O_6_, 98%), caffeic acid (C_9_H_8_O_4_, 98%), p-coumaric acid (C_9_H_8_O_3_, 95%), ferulic acid (C_10_H_10_O_4_, 98%), 2,4-dimethoxycinnamic acid (C_11_H_12_O_4_, 95–98%), artepillin C (C_27_H_32_O_5_, ≥95%), catechin (C_15_H_14_O_6_, ≥98%), gallic acid (C_7_H_6_O_5_, ≥98%), procyanidin B2 (C_30_H_26_O_12_, ≥95%), and pinocembrin (C_15_H_12_O_4_, ≥98%) were obtained from Sigma-Aldrich (Darmstadt, Germany)

Essential reagents including Folin–Ciocalteu’s phenol reagent (analytical grade), aluminum chloride (AlCl_3_·6H_2_O, ≥98%), sodium carbonate (Na_2_CO_3_, ≥99.5%), potassium acetate (CH_3_COOK, ≥99%), and 1,1-diphenyl-2-picrylhydrazyl (DPPH, C_18_H_12_N_5_O_6_, ≥95%) were supplied by Sigma-Aldrich (Darmstadt, Germany). Potassium bromide (KBr, 99%), zinc nitrate hexahydrate (Zn(NO_3_)_2_·6H_2_O, ≥98%), sodium hydroxide (NaOH, ≥98%), ethanol (C_2_H_5_OH, 99.9%), and methanol (CH_3_OH, 99.9%) were procured from Merck (Darmstadt, Germany).

Petri dishes used for microbial culturing were supplied by ISOLAB (Wertheim, Germany). Culture media including Mueller–Hinton agar (standardized formulation, microbiological grade), Mueller–Hinton broth (standardized formulation, microbiological grade), and Sabouraud Dextrose Agar (SDA, standard microbiological grade) were acquired from Oxoid Ltd. (Hampshire, UK). Antibiotic disks containing tetracycline (C_22_H_24_N_2_O_8_, ≥98%, 30 µg per disk) and fluconazole (C_13_H_12_F_2_N_6_O, ≥98%, 25 µg per disk) were purchased from Bioanalyse (Ankara, Turkey). The 0.5 McFarland turbidity standard (BaSO_4_ suspension, equivalent to 1.5 × 10^8^ CFU/mL, analytical grade) used for microbial standardization was obtained from Thermo Fisher Scientific (Waltham, MA, USA). The biomass of *A. platensis* was obtained from Hawaii *Arthrospira* Pacifica, Cyanotech Corp., located in Kailua-Kona, HI, USA.

### 2.2. Methods

#### 2.2.1. Extraction of *A. platensis*

The extraction of *A. platensis* was carried out according to the method described in [20]. In this process, approximately 10 g of dried *A. platensis* biomass were combined with 150 mL of an ethanol–water mixture (80:20 *v*/*v*) and stirred for 15 min. The mixture was then allowed to stand at room temperature for six hours. Afterward, it was filtered through Whatman No. 1 filter paper, and the filtrate was subjected to evaporation under reduced pressure at 50 °C and 70 rpm using a rotary evaporator (Heidolph Instrument GmbH & Co. KG, Schwabach, Germany) until complete removal of the solvent. The resulting extract was transferred into an airtight glass container and stored at 4 °C until further use.

#### 2.2.2. Determination of Total Phenolic Content (TPC), Total Flavonoid Content (TFC), and DPPH Radical Scavenging Activity

TPC of the extracts was measured using the Folin–Ciocalteu assay, as outlined by previous study [20]. A volume of 450 µL of the extract was combined with 2.25 mL of diluted Folin–Ciocalteu reagent (1:9, *v*/*v*) and shaken for 3 min at room temperature. Subsequently, 1.8 mL of sodium carbonate solution (75 g/L) was added, and the mixture was incubated in the dark at room temperature for 2 h. Absorbance was then recorded at 760 nm using a spectrophotometer. TPC results were expressed as milligrams of gallic acid equivalents per gram of the sample (mg GAE/g), based on a calibration curve prepared with standard gallic acid. All measurements were carried out in triplicate.

TFC was determined following the aluminum chloride colorimetric method described before [21]. In this procedure, 0.5 mL of the extract was mixed with 1.5 mL of 95% ethanol, 0.1 mL of 10% aluminum chloride solution, 0.1 mL of 1 M potassium acetate, and 2.8 mL of distilled water. After incubating the mixture at room temperature for one hour, absorbance was measured at 415 nm using a spectrophotometer. TFC values were calculated as milligrams of quercetin equivalents per gram of the sample (mg QE/g), using a quercetin standard curve. Each analysis was performed in triplicate.

The free radical scavenging activity was assessed using the DPPH (2,2-diphenyl-1-picrylhydrazyl) method, following the protocol previously described in [22] with some modifications. A 500 µL aliquot of the extract was added to 2.5 mL of a methanolic DPPH solution, adjusted to an initial absorbance of 0.800 ± 0.010 at 517 nm. The reaction mixture was incubated in the dark for 60 min, after which the absorbance was measured at 517 nm against a methanol blank. The percentage of DPPH radical scavenging activity was calculated using Equation (1):

DPPH Scavenging Activity% = (1 − [A sample/A blank]) × 100(1)

where A sample is the absorbance of the test sample and A blank is the absorbance of the control.

To determine EC_50_ values, various concentrations of the samples were tested to plot antiradical activity curves. All measurements were conducted in triplicate.

TPC, TFC, and DPPH radical scavenging activities of *A. platensis*-ZnO NPs were determined using the procedures previously described in this section. These methods were also applied to the *A. platensis*-ZnO NPs samples, with ethanol-based extraction used for sample preparation. Specifically, 10 mg of the dried *A. platensis*-ZnO NPs were suspended in 10 mL of 80% ethanol and subjected to sonication for 30 min to ensure proper dispersion and extraction of surface-bound bioactive compounds. The mixture was then centrifuged at 6000 rpm for 15 min at room temperature. The clear supernatant was carefully collected and used as the test solution for TPC, TFC, and DPPH assays. All measurements were performed in triplicate. Results were reported as milligrams of gallic acid equivalents per gram of sample (mg GAE/g) for TPC, milligrams of quercetin equivalents per gram (mg QE/g) for TFC, and percentage inhibition for DPPH radical scavenging activity. EC_50_ values were determined by plotting scavenging activity against concentration.

#### 2.2.3. HPLC Analysis

Extracts of *A. platensis* were prepared according to a previously described method [23] with minor modifications. Samples were centrifuged at 5000 rpm for 3 min, and the supernatant was filtered through a 0.45 µm PTFE syringe filter (Sigma-Aldrich, St. Louis, MO, USA). A 25 µL aliquot was injected into an HPLC system (Shimadzu SPD-20A, Kyoto, Japan) equipped with a C18 analytical column (4.6 × 250 mm, 5 µm; GL Sciences, Tokyo, Japan).

Phenolic compounds were separated using a two-solvent gradient elution: Solvent A (acetic acid–water, 2:98 *v*/*v*) and Solvent B (methanol–water, 50:50 *v*/*v*). The gradient began with 3% B, increased to 5% at 3 min, 20% at 20 min, 25% at 30 min, 30% at 40 min, 50% at 55 min, and 100% at 65 min, held for 10 min, and then re-equilibrated to the initial conditions. The flow rate was 1 mL/min, column temperature 30 °C, and detection wavelength 280 nm.

Phenolic compounds were identified by comparison with UV absorption spectra of standards, and concentrations were expressed in mg/L (ppm) using calibration curves.

#### 2.2.4. Synthesis of *A. platensis*-ZnO NPs

To prepare the extract, 10 g of dried *A. platensis* powder was mixed with 200 mL of 80% ethanol and heated at 80 °C for 1 h under continuous stirring. The mixture was then filtered and centrifuged at 15,000 rpm for 15 min to remove residues. The supernatant was subsequently stored for nanoparticle synthesis. The green synthesis of *A. platensis*-ZnO NPs was performed using a modified co-precipitation method [24]. Briefly, 25 mL of 0.05 M Zn(NO_3_)_2_·6H_2_O solution was mixed with 10 mL of the *A. platensis* extract and stirred at 80 °C for 1 h. The pH of the solution was adjusted between 8 and 12 by adding 0.2 M NaOH dropwise, followed by an additional hour of stirring. The resulting precipitate was washed several times with ultrapure deionized water and ethanol, then centrifuged at 15,000 rpm (EBA 20 Hettich, Tuttlingen, Germany). Two types of nanoparticle samples were prepared: one was dried overnight at 80 °C, and the other was calcinated at 400 °C for 3 h following initial drying. Both products were subsequently ground gently into fine powders. A control ZnO NPs sample was synthesized under identical conditions, excluding the algal extract.

#### 2.2.5. Physicochemical Characterization of *A. platensis*-ZnO NPs

To evaluate the structural and compositional properties of the biosynthesized ZnO NPs derived from *A. platensis*, a series of advanced analytical techniques were employed. Prior to characterization, the ZnO NPs underwent purification through repeated washing with distilled water and ethanol to eliminate residual biomolecules and unreacted algal components. The suspension was then centrifuged at 8000 rpm for 15 min. The purified nanoparticles were dried in a laboratory oven at 60 °C for 12 h and subsequently stored in airtight containers for further use. UV–Vis spectroscopy, 1 mL of the dried ZnO NPs sample was redispersed in distilled water and vortexed for 15 min using a Vortex-Genie 2 (Scientific Industries, Bohemia, NY, USA) to ensure a homogeneous suspension. The absorbance spectrum was recorded across the wavelength range of 200–400 nm using a UV–Vis spectrophotometer (UV-1800, Shimadzu Corporation, Kyoto, Japan).

FT-IR spectroscopy was conducted to identify surface functional groups and confirm interactions between *A. platensis* metabolites and the ZnO NPs surface. The dried powder was thoroughly mixed with potassium bromide (KBr), compressed into a pellet, and analyzed using a Perkin Elmer Spectrum 100 spectrometer (Rodgau, Germany) over the range of 4000–400 cm^−1^. Morphological features were examined using FE-SEM microscopy. A small quantity of dried ZnO nanopowder was spread onto a carbon-coated aluminum stub and coated with a thin layer of gold via sputtering to enhance conductivity. Imaging was performed using a Zeiss SIGMA VP-500 FE-SEM (Oberkochen, Germany) to observe particle shape and surface texture. The average particle size of the ZnO NPs was determined from FE-SEM micrographs using ImageJ software, version 1.8.0-112 (National Institutes of Health, Bethesda, MD, USA). For each sample, at least 100 individual particles were randomly selected from different fields of view, and their diameters were measured. The mean particle size was calculated as the arithmetic average ± standard deviation of these measurements. Elemental analysis was carried out using EDX spectroscopy coupled with the FE-SEM. The Oxford Instruments EDX detector (Abingdon, UK) was used to verify the elemental composition of the nanoparticles and confirm the successful incorporation of zinc and oxygen. All characterization experiments were performed in triplicate to ensure reproducibility and data reliability.

#### 2.2.6. Antimicrobial Activity Assessment

The antimicrobial properties of *A. platensis*-ZnO NPs were evaluated against three standard microbial strains obtained from the Microbiology Culture Collection at Gaziantep University: *S. aureus* ATCC 25923 (Gram-positive), *E. coli* ATCC 25922 (Gram-negative), and *C. albicans* ATCC 10231 (yeast). These strains were selected for their relevance in clinical infections and standardized response in susceptibility assays. Bacterial cultures were grown on nutrient agar, and the yeast was maintained on Sabouraud dextrose agar at 4 °C until use. To prepare the inocula, loopfuls of each microorganism were transferred to appropriate broths (Mueller–Hinton for bacteria, Sabouraud for fungi) and incubated at 37 °C for 24 h (*S. aureus*, *E. coli*) and 28 °C for 7 days (*C. albicans*) to achieve moderate turbidity. The resulting suspensions were adjusted to the 0.5 McFarland standard using sterile saline and subsequently diluted to 10^6^ CFU/mL for testing. For antimicrobial testing, *A. platensis*-ZnO NPs were dispersed in sterile distilled water at 10 mg/mL and sonicated for 15 min, while the crude *A. platensis* extract was prepared at the same concentration using 5% DMSO. Both solutions were sterilized by passage through 0.22 µm syringe filters. Quantitative analysis of microdilution antimicrobial activity was conducted using the broth method, as per CLSI guidelines [25] with slight modifications.

Serial two-fold dilutions of each test sample were prepared in 96-well microplates containing Mueller–Hinton broth (bacteria) or Sabouraud broth (fungi) and inoculated with 100 µL of microbial suspension. Negative controls contained broth with inoculum only, while sterility controls had broth without inoculum or test material. Following incubation (37 °C for 24 h for bacteria; 28 °C for 48 h for yeast), the minimum inhibitory concentration (MIC) was determined as the lowest concentration without visible turbidity. To establish the minimum bactericidal or fungicidal concentration (MBC/MFC), 10 µL from wells showing no growth was subcultured onto fresh agar plates and incubated again. The MBC/MFC was defined as the lowest concentration that completely inhibited colony formation. Qualitative evaluation was also performed using the agar well diffusion method [26]. Mueller–Hinton and Sabouraud dextrose agars were poured into sterile Petri dishes, and 100 µL of each standardized microbial suspension was spread evenly on the surface. Wells (6 mm diameter) were aseptically punched into the agar and filled with 100 µL of each test solution at different concentrations. Tetracycline (30 µg) and fluconazole (25 µg) from Bioanalyse (Ankara, Turkey) were used as positive controls. After incubation (37 °C for bacteria, 28 °C for yeast), zones of inhibition were measured in millimeters using a digital caliper. All tests—including MIC, MBC/MFC, and zone diameter measurements—were carried out in triplicate, and results were expressed as mean ± standard deviation (SD).

#### 2.2.7. Statistical Analysis

Data were analyzed using one-way analysis of variance (ANOVA), followed by Tukey’s post hoc test to identify significant differences among treatment groups. A *p*-value less than 0.05 was considered indicative of statistical significance.

## 3. Results

### 3.1. Determination of Total Flavonoid Content (TFC), Total Phenolic Content (TPC), and DPPH Radical Scavenging Activity

The TFC of *A. platensis* extract was found to be 0.85 ± 0.02 mg QE/g, as shown in Table 1. Meanwhile, the TFC of *A. platensis*-ZnO NPs synthesized at 80 °C decreased to 0.75 ± 0.03 mg QE/g and further decreased to 0.68 ± 0.05 mg QE/g when synthesized at 400 °C (Table 1). These decreases are statistically significant (*p* < 0.05).

Similarly, the TPC of *A. platensis* extract was measured at 1.80 ± 0.03 mg GAE/g. When incorporated into ZnO NPs, the TPC showed a decline, with values of 1.65 ± 0.04 mg GAE/g at 80 °C and 1.45 ± 0.06 mg GAE/g at 400 °C (Table 1). These differences are also statistically significant (*p* < 0.05).

Regarding DPPH radical scavenging activity, *A. platensis* extract exhibited an IC_50_ value of 12.0 ± 0.55 μg/mL. In comparison, *A. platensis*-ZnO NPs synthesized at 80 °C displayed a slightly higher IC_50_ value of 13.0 ± 0.60 μg/mL, while those synthesized at 400 °C showed the highest IC_50_ value of 14.0 ± 0.70 μg/mL. These differences are statistically significant (*p* < 0.05) (Table 1).

### 3.2. Phenolic Profile of A. platensis Extract

**Figure 1:**
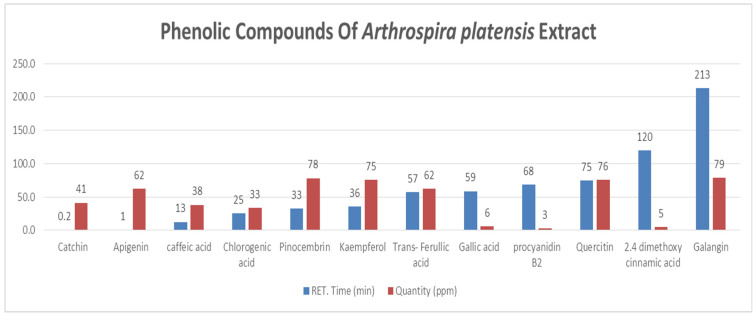
Phenolic Compounds of *A. platensis* Extract.

HPLC analysis of *A. platensis* extract led to the identification of 12 phenolic compounds (Figure 1). Among these, prominent flavonoids such as galangin (78.62 ± 3.93 ppm), pinocembrin (78.15 ± 3.91 ppm), and kaempferol (75.47 ± 3.77 ppm) were present in high concentrations, highlighting *A. platensis* as a rich source of bioactive flavonoids. Apigenin (62.41 ± 3.12 ppm) and trans-ferulic acid (62.28 ± 3.11 ppm) were present at moderate levels. In contrast, catechin (41.27 ± 2.06 ppm), gallic acid (6.16 ± 0.31 ppm), and procyanidin B2 (2.96 ± 0.15 ppm) were detected in lower concentrations, while 2,4-dimethoxy cinnamic acid (5.00 ± 0.25 ppm) was also identified among the minor compounds (Table 2).

### 3.3. Characterization of ZnO NPs

**Figure 2:**
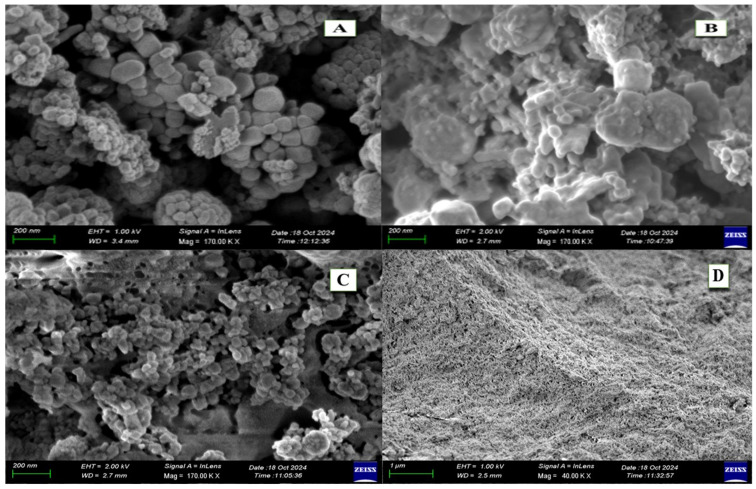
Morphological comparison of *A. platensis*–mediated ZnO NPs, pure ZnO NPs, and *A. platensis* extract. (**A**): 80 °C dried sample (45.2 nm); (**B**): 400 °C calcined sample (37.1 nm); (**C**): pure ZnO NPs (26.4 nm); (**D**): *A. platensis* extract (amorphous structures).

The FE-SEM analysis revealed clear morphological differences between the biosynthesized ZnO NPs, pure ZnO NPs, and the *A. platensis* extract (Figure 2). The biosynthesized ZnO NPs produced at 80 °C (Figure 2A) showed polydispersed, irregular granular structures with an average size of 45.2 ± 8.6 nm (n = 100), while the calcined sample at 400 °C (Figure 2B) exhibited more compact and angular nanoparticles averaging 37.1 ± 6.3 nm. In contrast, the pure ZnO NPs (Figure 2C) were predominantly uniform and smaller (26.4 ± 4.1 nm), while the *A. platensis* extract alone (Figure 2D) displayed amorphous, irregular structures.

**Figure 3:**
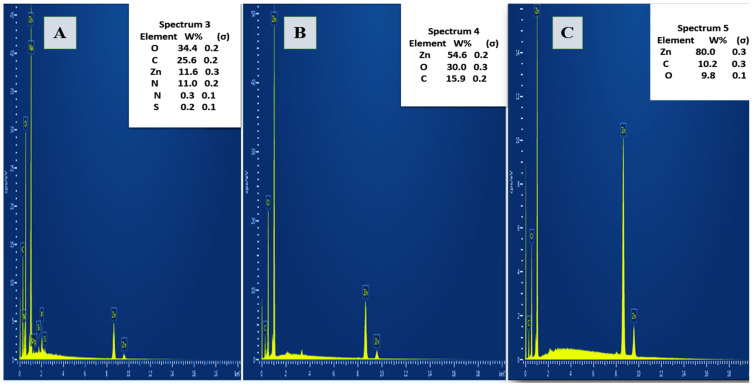
EDX Spectra of *A. platensis*-Mediated ZnO NPs Synthesized at 80 °C (**A**) and 400 °C (**B**) Pure ZnO NPs (**C**) Showing Elemental Composition Variation with Calcination Temperature.

The EDX analy sis presented in Figure 3 illustrates the elemental composition of *A. platensis*-mediated ZnO NPs synthesized at two different conditions: 80 °C (A) and 400 °C (B), along with pure ZnO NPs (C). In spectrum 3 the elemental composition of ZnO NPs synthesized at 80 °C shows a significant presence of carbon (34.4%), oxygen (25.6%), and zinc (18.8%), with additional minor elements such as nitrogen, sodium, phosphorus, sulfur, and magnesium. In contrast, the EDX spectrum of ZnO NPs synthesized at 400 °C (spectrum 4) reveals a markedly higher zinc content (54.0%) and lower carbon (15.9%), while oxygen levels remain relatively consistent (30.0%), and a similar pattern can be observed in the pure ZnO NPs sample (spectrum 5), which shows the highest zinc content (80.0%) with minimal oxygen (9.8%) and carbon

**Figure 4:**
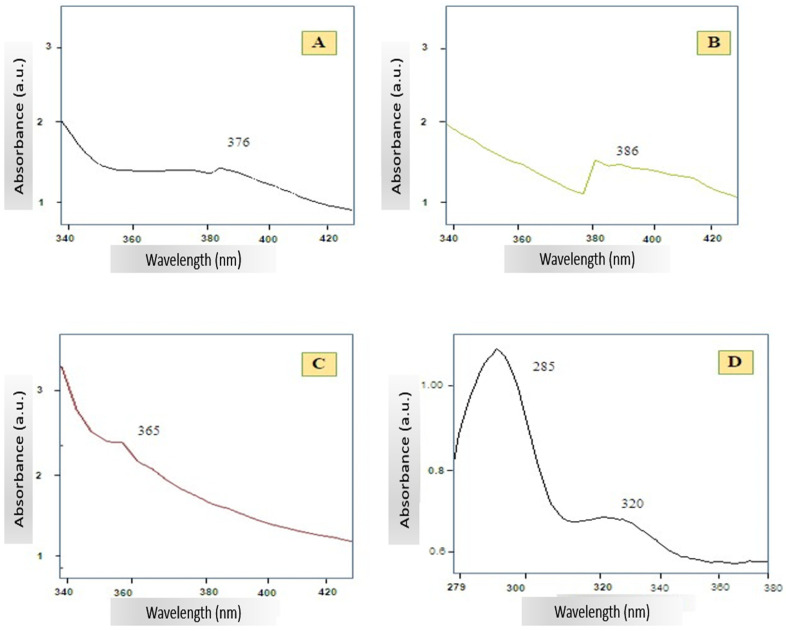
UV–Vis absorption spectra of *A. platensis*-mediated ZnO NPs synthesized at 80 °C (**A**) and 400 °C (**B**), pure ZnO NPs (**C**), and *A. platensis* extract (**D**).

The UV–Vis spectrum of *A. platensis*-mediated ZnO NPs, synthesized at two different temperatures 80 °C (A) and 400 °C (B) is shown in Figure 4. In both cases, a distinct excitonic absorption peak is observed in the UV region, with Spectrum A exhibiting maximum absorbance at 376 nm and Spectrum B displaying a clear peak at 386 nm. Such sharp absorption bands are characteristic of ZnO NPs and indicate strong excitonic transitions at room temperature. The spectrum of pure ZnO NPs (Figure 4C) exhibited a pronounced excitonic absorption peak at ~365 nm. Meanwhile, the *A. platensis* extract alone (Figure 4D) exhibited a strong absorption peak at approximately 295–300 nm, with a gradual decline into the 330–350 nm range.

**Figure 5:**
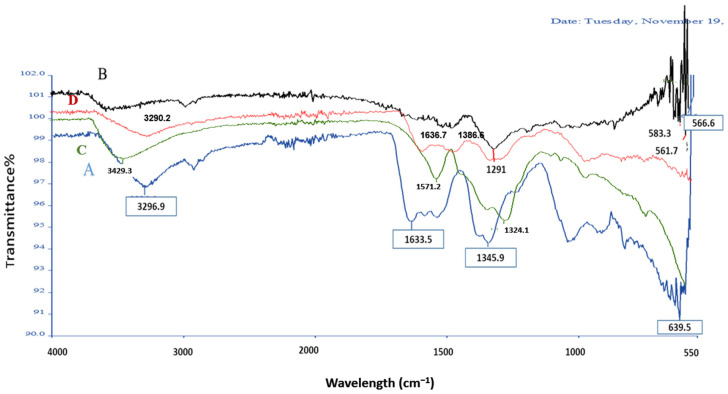
FT-IR spectra of *A. platensis*-mediated ZnO nanoparticles synthesized at two different temperatures: 80 °C ((**A**), blue line) and 400 °C ((**B**), black line), ((**C**), green line: *A. platensis* Extract), ((**D**), red line: pure ZnO NPs).

The FTIR spectra of *A. platensis*-mediated ZnO NPs (Figure 5) confirm the successful synthesis of ZnO NPs and provide insights into the role of biomolecules in stabilization. Both spectra (before and after calcination) display a strong absorption band near 470 cm^−1^, corresponding to Zn–O stretching vibrations, which is characteristic of ZnO NPs. This band confirms the formation of ZnO bonds. Before calcination (80 °C), several additional absorption peaks are observed, including a broad band at 3296–3400 cm^−1^ (O–H/N–H stretching), a band around 1634–1635 cm^−1^ (amide I, C=O stretching), and peaks near 1345–1385 cm^−1^ (C–N/C–O stretching). These indicate the presence of proteins, polysaccharides, and polyphenolic compounds from *A. platensis*.

In contrast, the FTIR spectrum of pure ZnO NPs (Figure 5D) showed predominantly sharp Zn–O stretching vibrations in the 560–580 cm^−1^ region, with minimal or no signals corresponding to organic functional groups. Meanwhile, the FTIR spectrum of *A. platensis* extract alone (Figure 5C) displayed characteristic biomolecular peaks, including a broad band at 3429 cm^−1^ (O–H/N–H stretching), a strong band around 1570–1640 cm^−1^ (amide I, C=O stretching), and additional peaks at 1290–1345 cm^−1^ (C–N/C–O stretching). These features confirm the abundance of proteins, polysaccharides, and polyphenols in the extract.

### 3.4. Antimicrobial Activity Results

The comparative analysis of MIC and MBC/MFC values for *A. platensis*-ZnO NPs synthesized at two different temperatures (80 °C and 400 °C), pure ZnO NPs, and extract against *S. aureus*, *E. coli*, and *C. albicans* highlights notable differences in antimicrobial efficacy (Table 3). For *S. aureus*, *A. platensis*-ZnO NPs showed strong antimicrobial activity, with MIC/MBC values of 16/16 µg/mL (80 °C) and 8/16 µg/mL (400 °C). In comparison, pure ZnO NPs recorded 32/32 µg/mL (80 °C) and 16/16 µg/mL (400 °C). The *A. platensis* extract exhibited inhibition with both MIC and MBC at 128 µg/mL. Against *E. coli*, *A. platensis*-ZnO NPs synthesized at 80 °C exhibited MIC/MBC values of 32/64 µg/mL, while those prepared at 400 °C showed 16/16 µg/mL. Pure ZnO NPs displayed 64/128 µg/mL (80 °C) and 32/32 µg/mL (400 °C), while the extract required 256/256 µg/mL to achieve inhibitory and bactericidal effects, respectively. In the case of *C. albicans*, *A. platensis*-ZnO NPs synthesized at 80 °C had MIC/MFC values of 64/128 µg/mL, whereas those calcined at 400 °C achieved 32/32 µg/mL, indicating markedly stronger antifungal activity at elevated temperature. Pure ZnO NPs were less effective, with MIC/MFC values of 128/128 µg/mL (80 °C) and 128/128 µg/mL (400 °C). The extract remained the least effective, with MIC/MFC values of 256/256 µg/mL.

The agar well diffusion results demonstrate that *A. platensis*–mediated ZnO NPs exhibited superior antimicrobial activity compared to both pure ZnO NPs and the crude *A. platensis* extract (Table 4). Among the tested microorganisms, the strongest inhibition zone was observed against *S. aureus* (23.5 ± 3.5 mm at 80 °C and 26.0 ± 4.1 mm at 400 °C), followed by *C. albicans* and *E. coli*. For *E. coli*, the inhibition zones measured 17.8 ± 2.3 mm for nanoparticles synthesized at 80 °C and 19.5 ± 2.1 mm at 400 °C, compared with 15.2 ± 2.8 mm for the extract and 10.5 ± 0.4 mm for pure ZnO NPs. For *C. albicans*, the inhibition zones were 18.2 ± 2.5 mm for nanoparticles synthesized at 80 °C and 20.0 ± 3.0 mm at 400 °C, compared with 14.0 ± 1.9 mm for the extract and 11.0 ± 0.6 mm for pure ZnO NPs.

## 4. Discussion

### 4.1. Determination of Total Phenolic Content (TPC), Total Flavonoid Content (TFC), and DPPH Radical Scavenging Activity

The results of total flavonoid and phenolic content showed that the synthesis of ZnO nanoparticles using *A. platensis* led to a noticeable reduction in these bioactive compounds, particularly at higher synthesis temperatures. The reduction in flavonoid content in the nanoparticle composites could be due to heat-induced degradation during the synthesis process or possibly due to the limited interaction between the flavonoids and the nanoparticle surface at higher temperatures [18,19]. For example, previous study reported a flavonoid content of 29.65 ± 6.52 mg quercetin equivalent (QE)/g in a bioactive fraction of *A. platensis*, which is significantly higher than the 0.85 mg QE/g found in the current study. This highlights some variation in flavonoid content depending on the extraction methods and fractions used [27].

Additionally, another study focused on the biosynthesis of ZnO NPs from *A. platensis* but did not explicitly mention the flavonoid content. However, it suggests that the synthesis of nanoparticles using *A. platensis* can influence its biochemical composition, including potential changes to flavonoid levels [8]. Similarly, scientists examined the impact of silver nanoparticles on *A. platensis* and observed a 79% reduction in protein content upon exposure. Although the study focused primarily on protein levels, the findings also suggest potential implications for the broader phytochemical profile of *A. platensis*, including possible effects on flavonoid stability and retention. [28]. These studies indicate that the synthesis conditions and the presence of ZnO NPs can influence the flavonoid content of *A. platensis*, although direct comparisons to the current study are limited. The interaction between *A. platensis* and ZnO NPs may result in variations in the retention or degradation of bioactive compounds, including flavonoids, depending on synthesis temperatures and other factors.

The TPC of *A. platensis* extract showed a clear reduction after incorporation into ZnO nanoparticles, with progressively lower values observed at higher synthesis temperatures. This suggests that the incorporation of ZnO NPs reduces the phenolic content, likely due to interactions between phenolic compounds and the nanoparticle surface or heat-induced degradation during synthesis [29]. Despite this reduction, the ZnO NP composites still retain a substantial amount of phenolic compounds, which may contribute to their stability and bioactivity in various applications.

The results for DPPH radical scavenging activity indicate a clear reduction in antioxidant capacity with increasing synthesis temperature, likely due to the thermal degradation of phenolic and flavonoid compounds that play a key role in radical scavenging. At lower synthesis temperatures, more heat-sensitive phytochemicals are preserved on the nanoparticle surface, enabling these compounds to act synergistically with the natural ROS-scavenging ability of ZnO NPs. In contrast, at higher calcination temperatures, the loss of these organic capping agents leads to a greater reliance on the comparatively weaker antioxidant potential of pure ZnO [7,18].

Despite this reduction, the nanoparticle composites still display notable antioxidant activity, suggesting that even the residual phytochemicals, in combination with ZnO’s intrinsic properties, can provide a meaningful ROS-scavenging effect. Comparable patterns have been observed in plant-mediated ZnO NP studies, where higher synthesis temperatures lower total phenolic and flavonoid contents, thereby reducing antioxidant activity [12,19]. However, exceptions exist; for example, scientists observed higher antioxidant activity in Spirulina-capped ZnO NPs calcined at 600 °C compared to lower-temperature counterparts, indicating that the impact of calcination temperature can depend on the type of biomass used, the stability of capping agents, and the synthesis method. These observations highlight the importance of optimizing synthesis conditions to balance nanoparticle crystallinity with the retention of bioactive compounds [29]. Such optimization is particularly relevant for applications in food preservation, nutraceuticals, and pharmaceuticals, where both stability and bioactivity are essential.

### 4.2. HPLC Analysis of Phenolics

The HPLC analysis highlights the diverse phenolic profile of *A. platensis*, emphasizing the predominance of flavonoids alongside several key phenolic acids. This composition is consistent with previous studies reporting the presence of bioactive flavonoids in cyanobacteria-derived extracts, which are known for their strong antioxidant and antimicrobial properties [2,20]. Phenolic acids such as chlorogenic acid and caffeic acid are widely recognized for their anti-inflammatory, cardioprotective, and neuroprotective properties, and their presence supports the potential of *A. platensis* in nutraceutical and therapeutic applications [13].

Moreover, moderate levels of specific flavonoids, including compounds like apigenin and trans-ferulic acid, may contribute to cellular protection through their roles in modulating oxidative pathways and reducing reactive oxygen species (ROS) [30]. Even the minor phenolic constituents, such as catechin, gallic acid, procyanidin derivatives, and cinnamate compounds, likely enhance the overall antioxidant capacity and provide additional functional benefits, including antimicrobial and UV-protective properties [31]. These observations are in line with earlier reports describing the wide range of phenolics present in *A. platensis* and their involvement in oxidative damage protection and immune function enhancement [10,12,13]. Overall, the phenolic composition observed in this study underscores the value of *A. platensis* as a rich natural source of antioxidants with potential applications in the food, pharmaceutical, and cosmetic industries.

### 4.3. Structural and Morphological Characterization

The results of the FE-SEM analysis showed clear morphological differences between the biosynthesized *A. platensis*-ZnO NPs, pure ZnO NPs, and the *A. platensis* extract, with changes in particle shape and size observed between low and high synthesis temperatures. These observations suggest that biomolecules present in the algal extract likely act as both reducing and stabilizing agents during nanoparticle synthesis, resulting in heterogeneous nucleation and capping processes that contribute to greater polydispersity compared with pure ZnO NPs. Comparable findings have been documented in several recent studies. For instance, an investigation on Arabic gum–mediated ZnO NPs showed that increasing calcination temperature led to a reduction in particle size and a transition from loosely aggregated to more compact and crystalline structures [32]. Similarly, a comprehensive review on green-synthesized ZnO NPs emphasized that thermal treatment plays a decisive role in determining nanoparticle morphology, with higher temperatures yielding smaller, denser, and more crystalline particles [33]. Other studies also fall within a similar size range. A recent report observed spherical ZnO NPs synthesized through plant-mediated methods with average diameters between 30 and 40 nm, confirming that the 37 nm size obtained after calcination in the present study is well within the commonly reported nanoscale range [34]. Likewise, earlier works demonstrated that calcination at elevated temperatures often results in improved crystallinity and slight particle size reduction compared to low-temperature or oven-dried samples [7,18].

The EDX analysis showed distinct differences in the elemental composition of the biosynthesized ZnO NPs at different synthesis temperatures compared with the pure ZnO NPs, particularly in carbon and zinc content. The elevated carbon levels at lower synthesis temperatures are attributable to the presence of organic phytochemicals from the *A. platensis* extract, such as polysaccharides, flavonoids, and proteins, which function as reducing and stabilizing agents, thereby capping the nanoparticle surface. At higher calcination temperatures, the marked increase in zinc content and reduction in carbon indicate thermal degradation and removal of these organic residues, leading to purer, more crystalline ZnO NPs with fewer surface bound bio-organic compounds [19,32]. A previous study using Moringa oleifera seed extract yielded 86.79% Zn and only 2.73% C, underscoring the effect of synthesis temperature and plant extract type on elemental composition [35]. Similarly, ZnO NPs synthesized with *Cayratia pedata* leaf extract showed higher zinc content and lower organic residue, aligning with the profile observed in the 400 °C sample. These findings emphasize the critical role of both biomass source and thermal treatment in tuning the surface composition and purity of ZnO NPs [36]. The high carbon content at lower synthesis temperatures may be advantageous for applications requiring surface functionality, such as drug delivery or antimicrobial coatings, whereas higher zinc purity at elevated temperatures may benefit applications requiring enhanced optical or electronic properties [6,7,19,29].

The UV–Vis analysis demonstrated clear differences in the spectral profiles of the biosynthesized ZnO NPs, pure ZnO NPs, and the *A. platensis* extract, particularly in the position and intensity of the excitonic absorption peaks. The biosynthesized ZnO NPs exhibited characteristic UV absorption bands typical of ZnO NPs, with slight shifts depending on synthesis temperature. These spectral shifts are commonly attributed to differences in particle size, crystallinity, and the presence of residual capping agents from biological extracts. A small red shift at higher synthesis temperatures may indicate improved crystalline quality or aggregation, as elevated temperatures often lead to the removal of organic capping layers [24,26]. These observations are in line with earlier studies reporting excitonic absorption features for ZnO NPs around 368–376 nm at room temperature [37,38,39,40]. Green-synthesized ZnO systems typically show broader bands between 330 and 460 nm, with peak positions dependent on synthesis conditions and the nature of biological reducing agents [38,39]. The pure ZnO NPs displayed a sharper and more intense excitonic peak, consistent with chemically synthesized ZnO NPs and reflecting their more uniform structure and surface chemistry. In contrast, the *A. platensis* extract exhibited a distinct absorption band in the 295–300 nm region, characteristic of aromatic amino acids, proteins, and phenolic compounds such as flavonoids and phycocyanin [11]. The absence of a ZnO-specific excitonic peak confirms that this spectrum represents the algal extract alone. These biomolecules play a central role in the biosynthesis process by acting as reducing and stabilizing agents, ultimately influencing the optical properties of the resulting nanoparticles. Together, these spectral differences provide insight into how biological components and synthesis temperature affect nanoparticle optical behavior, underscoring the role of algal biomolecules in shaping the excitonic features and surface chemistry of green-synthesized ZnO nanoparticles.

Moreover, the results of FTIR analysis showed clear differences in the spectral features of the biosynthesized ZnO NPs, pure ZnO NPs, and the *A. platensis* extract, revealing distinct functional groups and bonding patterns before and after calcination. The biosynthesized ZnO NPs exhibited a strong absorption band near 470 cm^−1^, corresponding to Zn–O stretching vibrations, which is characteristic of ZnO nanoparticles and consistent with previous studies [29,41]. Before calcination (80 °C), several additional absorption peaks were associated with proteins, polysaccharides, and polyphenolic compounds from *A. platensis*, which act as capping and stabilizing agents for the nanoparticles. Similar findings have been reported in plant-mediated ZnO NPs, where phytochemicals such as flavonoids and alkaloids were responsible for surface stabilization [4,18]

After calcination at 400 °C, many of the organic functional group peaks were reduced or disappeared, leaving mainly Zn–O vibrations and weak residual signals. This observation aligns with previous studies demonstrating that calcination effectively eliminates biomolecule-related FTIR bands such as O–H and C–H stretching, resulting in the prominence of Zn–O stretching peaks typical of crystalline ZnO. For instance, one study recorded a marked reduction in the extract’s O–H peaks (3317 cm^−1^) post-ZnO formation, along with new Zn–O signatures in the 500–800 cm^−1^ region [42]. Similarly, another study found that ZnO NPs calcined at 500 °C retained only sharp Zn–O vibrations, while other organic functional group bands diminished significantly [43]. Overall, these results confirm that biomolecules from *A. platensis* extract contribute to the green synthesis of ZnO NPs by reducing and stabilizing zinc ions, while calcination enhances crystallinity and purity. This dual role of biological capping agents and subsequent calcination aligns with previous findings in phyto- and microalgae-mediated nanoparticle synthesis [7,29].

In contrast, the FTIR spectrum of pure ZnO NPs displayed sharp Zn–O stretching vibrations with minimal signals corresponding to organic functional groups, indicating higher purity and crystallinity compared with the biosynthesized samples. Meanwhile, the spectrum of the *A. platensis* extract alone exhibited characteristic biomolecular peaks associated with hydroxyl, amide, and C–N/C–O stretching vibrations. These features confirm the abundance of proteins, polysaccharides, and polyphenols in the extract, which play a critical role in nanoparticle reduction and stabilization during green synthesis [5,13,18].

Overall, these results confirm that biomolecules from *A. platensis* extract contribute to the green synthesis of ZnO NPs by reducing and stabilizing zinc ions, while calcination enhances crystallinity and purity. The comparison with pure ZnO NPs and the extract further emphasizes the dual role of biological capping agents in nanoparticle formation and the impact of calcination in minimizing organic residues, consistent with previous findings in phyto and microalgae mediated nanoparticle synthesis.

### 4.4. Antimicrobial Activity

#### 4.4.1. MIC and MBC/MFC Results

The MIC and MBC/MFC results demonstrate that antimicrobial activity varies depending on the synthesis temperature and the nature of the sample-biosynthesized ZnO NPs, pure ZnO, or the extract., particularly against Gram-positive and Gram-negative bacteria. These patterns provide important insights into how structural and compositional differences impact antibacterial activity. The slightly lower MIC at 400 °C compared to 80 °C against *S. aureus* suggests that higher crystallinity and the removal of organic residues at elevated temperatures may improve antibacterial performance against Gram-positive bacteria. This observation is consistent with previous finding, which demonstrated that lower calcination temperatures significantly enhance the antibacterial activity of ZnO NPs against *S. aureus*. These studies attributed the improved inhibitory effect to reduced crystallinity and the development of more favorable rod-like morphologies, which promote stronger interactions with bacterial cell membranes [19].

For *E. coli*, the improved MIC/MBC profile at 400 °C indicates that calcination enhances antibacterial efficacy by bringing MBC values closer to MIC, reflecting a stronger bactericidal effect. These results suggest that smaller particle size and the presence of bio-organic capping agents at 80 °C enhance surface reactivity and ROS generation, which is particularly critical for penetrating the outer membrane of Gram-negative bacteria. A recent study supports this, showing that ZnO NPs calcined at lower temperatures exhibited larger particle sizes removal, while those heat-treated at higher temperatures showed reduced antibacterial activity against *E. coli*-attributed to changes in surface area and morphology that impair reactive surface interactions [44].

The MIC/MFC results for *C. albicans* showed notably stronger antifungal activity for *A. platensis*-ZnO NPs synthesized at 400 °C compared to 80 °C and to both the pure ZnO and extract samples. This enhanced activity is likely due to the removal of organic capping agents during calcination, which exposes more reactive ZnO surfaces. These findings are consistent with a previous study reporting that chemically synthesized ZnO NPs exhibit strong antifungal effects through direct membrane disruption and oxidative stress, whereas biologically synthesized ZnO shows more moderate activity due to the presence of capping agents [45]. Importantly, the retention of significant antifungal potency by *A. platensis*-ZnO NPs compared with the extract alone suggests a synergistic effect between ZnO and bioactive metabolites. This observation aligns with earlier studies that highlighted size dependent antimicrobial activity of ZnO NPs and emphasized the role of both particle size and microbial cell wall structure in determining susceptibility [7,46].

Altogether, these findings indicate that *A. platensis*–mediated synthesis enhances the antifungal performance of ZnO NPs, with activity influenced by both calcination temperature and microbial type, making these nanoparticles a versatile antimicrobial platform that integrates the intrinsic properties of ZnO with the bioactivity of algal metabolites.

#### 4.4.2. Agar Well Diffusion Method Results

The inhibition zone results showed that *A. platensis*-ZnO NPs exhibited stronger antimicrobial activity than both pure ZnO NPs and the extract, with the highest inhibition observed against *S. aureus*, followed by *C. albicans* and *E. coli*. Notably, nanoparticles calcined at 400 °C consistently produced larger inhibition zones than those synthesized at 80 °C, indicating that calcination improves antimicrobial performance.

The enhanced activity observed against *S. aureus* aligns with previous findings showing that Gram-positive bacteria are generally more susceptible to ZnO NPs than Gram-negative bacteria [46]. This difference is attributed to variations in cell wall architecture, where the thick peptidoglycan layer of Gram-positive bacteria facilitates nanoparticle adhesion and ROS-mediated damage, whereas the outer membrane of Gram-negative organisms offers partial protection. Similarly, another study reported that smaller, well-crystallized ZnO NPs exhibit greater antimicrobial activity, consistent with the higher inhibition zones observed for the 400 °C samples [29]. This is further supported by recent work, a study of PEGylated ZnO NPs, demonstrating that ZnO NPs with high crystallinity, confirmed via XRD and FTIR, exhibit enhanced antibacterial activity and elevated ROS production, implying that structural integrity and reduced surface contamination significantly improve effectiveness [47]. Likewise, a previous study confirmed that calcination reduces organic residues and exposes more reactive ZnO facets, boosting antibacterial efficacy [48]. Another study also observed that smaller, highly crystalline ZnO NPs inhibited *E. coli* more effectively through enhanced ROS and Zn^2+^ ion release. In particular, detailed mechanistic investigations using *Campylobacter jejuni* as a model organism demonstrated that ZnO NPs generated significant amounts of reactive oxygen species, including superoxide and hydroxyl radicals, which caused oxidative stress and damage to cell membranes, proteins, and DNA [49]. Transmission electron microscopy revealed that the nanoparticles adhered to and disrupted the bacterial cell surface, leading to leakage of intracellular contents. The study further confirmed that Zn^2+^ ion dissolution from the ZnO lattice contributed to membrane destabilization and impaired bacterial metabolism, amplifying the overall antibacterial effect. These findings provide strong evidence that particle size and crystallinity are critical determinants of antimicrobial performance, since smaller, well-crystallized ZnO NPs present larger surface areas for ROS generation and more efficient ion release. Collectively, these results support our conclusion that the lower MIC values observed at 400 °C in our study reflect higher crystallinity and reduced organic residues, which together strengthen antibacterial activity against Gram-positive bacteria such as *S. aureus* [46].

The antifungal inhibition results clearly show that *A. platensis*-ZnO NPs are more effective than both pure ZnO NPs and the extract, with the calcined nanoparticles displaying the highest antifungal efficacy. These findings are consistent with previous studies that emphasized the ability of ZnO NPs to disrupt fungal cell membranes through oxidative stress and metal ion release [50]. The enhanced antifungal effect also suggests that phytochemicals present in *A. platensis* act synergistically during nanoparticle synthesis, improving stability and surface reactivity [50].

The moderate antimicrobial activity of the crude *A. platensis* extract is supported by earlier reports on cyanobacterial metabolites containing phenolic and proteinaceous compounds with antibacterial and antifungal properties [51]. When incorporated into ZnO NPs, however, the antimicrobial activity increased significantly, confirming the contribution of bio-capping agents to improved nanoparticle–microbe interactions. Comparable observations were reported by another study, which found that biogenic ZnO NPs retained considerable antimicrobial potency relative to chemically synthesized counterparts [16].

Although the positive controls (tetracycline and fluconazole) exhibited the highest inhibition zones overall, the performance of the *A. platensis*–ZnO NPs, especially at 400 °C against *S. aureus*, was comparable (26.0 mm vs. 30.5 mm). It is important to note, however, that organic residues can remain after calcination. Thermogravimetric analysis (TGA) studies have shown that ZnO NPs synthesized at low temperatures (≤80 °C) retain significant organic residues, as indicated by high weight loss below 300 °C, whereas nanoparticles calcined at 400 °C exhibit minimal weight loss, reflecting the removal of most biomolecules [48]. Additional TGA and FTIR analyses have demonstrated that ZnO NPs heated to 400–500 °C display sharper and more intense Zn–O stretching peaks in FTIR spectra while bands corresponding to hydroxyl and organic groups diminish, indicating fewer surface residues. Corresponding TGA/DSC curves confirm that high-temperature calcined samples undergo only minor mass losses compared with low-temperature samples, supporting the conclusion that organic contaminants are largely removed at 400–500 °C [52]. Another study likewise reported that as calcination temperature increases, weight losses associated with organic biomolecules diminish, indicating higher purity and crystallinity of the ZnO NPs [53]. These observations suggest that while trace residues may persist at 400 °C, their levels are substantially reduced compared to 80 °C, reinforcing the interpretation that calcination improves nanoparticle purity and strengthens antimicrobial performance.

Moreover, the strong inhibition zones obtained against both bacterial and fungal strains demonstrate the potential of *A. platensis*–mediated ZnO NPs as effective antimicrobial agents, particularly relevant in the context of rising antibiotic resistance. In conclusion, the results confirm that biogenically synthesized ZnO NPs possess superior antimicrobial properties compared to pure ZnO NPs and crude extract. This is consistent with the broader body of literature and underscores the value of biogenic synthesis in producing nanoparticles with enhanced biological functionality. Future investigations should focus on clarifying the molecular mechanisms of action in greater detail, assessing cytotoxicity profiles, and evaluating practical applications in medical, pharmaceutical, and food preservation fields.

## 5. Conclusions

This study shows that *A. platensis* extract can serve as a green reducing and stabilizing agent for the synthesis of ZnO NPs and that calcination temperature strongly shapes their structure and function. Drying at 80 °C preserved more bioactive capping, yielding higher phenolic and flavonoid content and stronger antioxidant capacity, whereas calcination at 400 °C produced smaller, more crystalline particles with reduced organic residues and consistently superior antibacterial and antifungal activity, most notably against *S. aureus*. The combined evidence supports the view that crystallinity, surface cleanliness, and particle size collectively play a decisive role in determining antimicrobial performance in these biogenic ZnO systems.

Although the results are promising, several limitations should be acknowledged to provide context for their interpretation. The antimicrobial assays relied on commercial reference strains (ATCC), which are appropriate for standardization but do not capture the genetic and phenotypic diversity of clinical isolates; differences in membrane composition, efflux activity, and resistance determinants may influence susceptibility. The investigation also focused on only two thermal regimes, 80 °C and 400 °C, which restricts insight into the broader structure–activity relationship across intermediate and higher calcination temperatures. Moreover, the mechanistic role of reactive oxygen species generation and Zn^2+^ release was inferred rather than directly measured, and the assays did not extend to time-kill kinetics, biofilm models, or potential synergistic interactions with conventional antibiotics. In addition, issues related to safety and translation remain unexplored, as cytotoxicity to mammalian cells, hemocompatibility, long-term stability, and reproducibility across production batches were not assessed.

Building on these limitations, future research should widen the calcination range to include intermediate and higher temperatures, thereby clarifying how structural transitions affect biological activity. Expanding testing to clinical isolates and biofilm models will provide a more realistic understanding of antimicrobial potential in clinical contexts. Incorporating direct quantification of ROS and Zn^2+^ release under assay conditions, along with advanced surface characterization techniques such as XPS and detailed electron microscopy, will strengthen mechanistic explanations. Parallel evaluation of cytotoxicity and hemocompatibility will be necessary to establish a safety profile, while studies on nanoparticle stability, reproducibility, and synergy with antibiotics will help determine translational viability. Taken together, the present findings highlight *A. platensis*–mediated ZnO NPs as promising eco-friendly antimicrobial candidates, and continued investigation along these lines will be essential to validate both their effectiveness and safety for applications in medicine, pharmaceuticals, and food preservation.

## Figures and Tables

**Table 1:** Total Flavonoid Content (TFC), Total Phenolic Content (TPC), and DPPH Radical Scavenging Activity of *A. platensis* Extract and *A. platensis*-ZnO NPs Synthesized at Different Temperatures.

Parameter	Unit	*A. platensis* Extract	*A. platensis*-ZnO NPs (80 °C Synthesis)	*A. platensis*-ZnO NPs (400 °C Synthesis)
Total Flavonoid Content	mg QE/g	0.85 ± 0.02 ^a^	0.75 ± 0.03 ^b^	0.68 ± 0.05 ^b^
Total Phenolic Content	mg GAE/g	1.80 ± 0.03 ^a^	1.65 ± 0.04 ^b^	1.45 ± 0.06 ^b^
DPPH Scavenging Activity	μg/mL	12.0 ± 0.55 ^a^	13.0 ± 0.60 ^b^	14.0 ± 0.70 ^b^

mg QE/g represents the amount of quercetin equivalent per gram of the sample, and mg GAE/g represents the amount of gallic acid equivalent per gram of the sample. The values are expressed as the mean ± standard deviation (SD). Statistically significant differences (*p* < 0.05) within the same row are indicated by different superscript letters.

**Table 2:** Phenolic content of *A. platensis*.

Phenolic Compounds	RET. Time	Quantity (ppm)
Chlorogenic acid	25.04	33.27 ± 1.66
caffeic acid	12.62	38.10 ± 1.90
Apigenin	0.57	62.41 ± 3.12
Galangin	213.40	78.62 ± 3.93
Trans-Ferullic acid	57.32	62.27 ± 3.11
Kaempferol	35.63	75.47 ± 3.77
2.4 dimethoxy cinnamic acid	119.56	5.00 ± 0.25
Pinocembrin	32.57	78.15 ± 3.91
Gallic acid	58.79	6.16 ± 0.31
procyanidin B2	68.46	2.96 ± 0.15
Quercitin	74.96	75.82 ± 3.79
Catchin	0.15	41.27 ± 2.06

**Table 3:** Minimum inhibitory concentration (MIC) and minimum bactericidal/fungicidal concentration (MBC/MFC) of *A. platensis* extract, *A. platensis*-mediated ZnO nanoparticles (80 °C and 400 °C), and pure ZnO NPs against *S. aureus*, *E. coli*, and *C. albicans* (µg/mL, mean ± SD).

Microorganism	Agent	MIC (µg/mL)	MBC/MFC (µg/mL)
*S. aureus*	*A. platensis*-ZnO NPs (80 °C)	16.00 ± 0.00 ^b^	16.00 ± 0.00 ^b^
	*A. platensis*-ZnO NPs (400 °C)	8.00 ± 0.00 ^b^	16.00 ± 0.00 ^b^
	Pure ZnO NPs (80 °C)	32.00 ± 0.00 ^a^	32.00 ± 0.00 ^a^
	Pure ZnO NPs (400 °C)	16.00 ± 0.00 ^b^	16.00 ± 0.00 ^b^
	*A. platensis* extract	128.00 ± 0.00 ^c^	128.00 ± 0.00 ^c^
*E. coli*	*A. platensis*-ZnO NPs (80 °C)	32.00 ± 0.00 ^b^	64.00 ± 0.00 ^b^
	*A. platensis*-ZnO NPs (400 °C)	16.00 ± 0.00 ^b^	16.00 ± 0.00 ^b^
	Pure ZnO NPs (80 °C)	64.00 ± 0.00 ^b^	128.00 ± 0.00 ^b^
	Pure ZnO NPs (400 °C)	32.00 ± 0.00 ^b^	32.00 ± 0.00 ^b^
	*A. platensis* extract	256.00 ± 0.00 ^c^	265.00 ± 0.00 ^c^
*C. albicans*	*A. platensis*-ZnO NPs (80 °C)	64.00 ± 0.00 ^b^	128.00 ± 0.00 ^b^
	*A. platensis*-ZnO NPs (400 °C)	32.00 ± 0.00 ^b^	32.00 ± 0.00 ^b^
	Pure ZnO NPs (80 °C)	128.00 ± 0.00 ^a^	128.00 ± 0.00 ^a^
	Pure ZnO NPs (400 °C)	128.00 ± 0.00 ^a b^	128.00 ± 0.00 ^a b^
	*A. platensis* extract	256.00 ± 0.00 ^c^	256.00 ± 0.00 ^c^

Values are expressed as mean ± SD. Different superscript letters indicate significant differences (*p* < 0.05) among treatments for each microorganism.

**Table 4:** Inhibition Zone Diameters (mm) of Pure ZnO NPs, *A. platensis* Extract, and *A. platensis*-ZnO NPs (80 °C and 400 °C) against Test Microorganisms.

Microorganism	Pure ZnO NPs	*A. platensis* Extract	*A. platensis*-ZnO NPs (80 °C)	*A. platensis*-ZnO NPs (400 °C)	Positive Control
*E. coli*	10.5 ± 0.4 ^c^	15.2 ± 2.8 ^b^	17.8 ± 2.3 ^b^	19.5 ± 2.1 ^a^	22.0 ± 3.1 ^a^ (Tetracycline)
*C. albicans*	11.0 ± 0.6 ^c^	14.0 ± 1.9 ^b^	18.2 ± 2.5 ^b^	20.0 ± 3.0 ^a^	26.0 ± 3.5 ^a^ (Fluconazole)
*S. aureus*	13.0 ± 1.1 ^c^	20.0 ± 4.2 ^b^	23.5 ± 3.5 ^b^	26.0 ± 4.1 ^a^	30.5 ± 4.0 ^a^ (Tetracycline)

Values are expressed as mean ± SD. Different superscript letters indicate significant differences (*p* < 0.05) among treatments for each microorganism.

## Data Availability

The data presented in this study are available on request from the corresponding author.

## References

[1] Murray C.J., Ikuta K.S., Sharara F., Swetschinski L., Aguilar G.R., Gray A., Han C., Bisignano C., Rao P., Wool E. (2022). Global burden of bacterial antimicrobial resistance: A systematic analysis. Lancet.

[2] Butler M.S., Henderson I.R., Capon R.J., Blaskovich M.A.T. (2023). Antibiotics in the clinical pipeline as of December 2022. J. Antibiot..

[3] Smith R.A., Mikanatha N.M., Read A.F. (2015). Antibiotic resistance: A primer and call to action. Health Commun..

[4] Mousa S.A., Wissa D.A., Hassan H.H., Ebnalwaled A.A., Khairy S.A. (2024). Enhanced photocatalytic activity of green synthesized zinc oxide nanoparticles using low-cost plant extracts. Sci. Rep..

[5] Lebaka V.R., Ravi P., Reddy M.C., Thummala C., Mandal T.K. (2025). Zinc oxide nanoparticles in modern science and technology: Multifunctional roles in healthcare, environmental remediation, and industry. Nanomaterials.

[6] El-belely E.F., Farag M.M.S., Said H.A., Amin A.S., Azab E., Gobouri A.A., Fouda A. (2021). Green synthesis of zinc oxide nanoparticles using *Arthrospira platensis* and evaluation of their biomedical activities. Nanomaterials.

[7] Zahra S., Qaisa S., Sheikh A., Bukhari H., Amin C.A. (2022). Effect of calcination temperature on the structure and morphology of zinc oxide nanoparticles synthesized by base-catalyzed aqueous sol-gel process. Eur. J. Chem..

[8] Abo-Shanab W.A., Elsilk S.E., Afifi S.S., El-Shenody R.A. (2025). Ecofriendly biosynthesis of zinc oxide nanoparticles from *Arthrospira platensis* and their assessment for antimicrobial, antibiofilm, anticancer potency and alleviation of copper stress in *Vicia faba* L.. J. Soil Sci. Plant Nutr..

[9] Galedari S., Teimouri M. (2020). Study of the physicochemical properties and anti-biofilm effects of synthesized zinc oxide nanoparticles using *Artemisia* plant. Int. J. Basic Sci. Med..

[10] Banoee M., Seif S., Nazari Z.E., Jafari-Fesharaki P., Shahverdi H.R., Moballegh A., Shahverdi A.R. (2010). ZnO Nanoparticles Enhanced Antibacterial Activity of Ciprofloxacin against *Staphylococcus aureus* and *Escherichia coli*. J. Biomed. Mater. Res. B Appl. Biomater..

[11] Gentscheva G., Angelov G., Dobreva D. (2023). Application of *Arthrospira platensis* for medicinal purposes and the food industry: A review of the literature. Life.

[12] Al-Wathnani H. (2012). Antibacterial activities of the extracts of cyanobacteria and green algae isolated from desert soil in Riyadh, Kingdom of Saudi Arabia. Afr. J. Biotechnol..

[13] Kelebek H., Uzlasir T., Kubra H. (2025). Bioactive compounds and health benefits of *Arthrospira platensis* and *Chlorella vulgaris*: A comprehensive review. Food Nutr..

[14] Lafarga T., Fernández-Sevilla J.M., González-López C., Acién-Fernández F.G. (2020). *Spirulina* for the food and functional food industries. Food Res. Int..

[15] Agarwal H., Venkat Kumar S., Rajeshkumar S. (2017). A review on green synthesis of zinc oxide nanoparticles–an eco-friendly approach. Resour. Technol..

[16] Ahmed N.A., Othman A.S. (2024). Green fabrication of ZnO nanoparticles via *Spirulina platensis* and its efficiency against biofilm-forming pathogens. Microb. Cell Fact..

[17] Kaewsaenee J., Singhaset M.T., Roongraung K., Kemacheevakul P., Chuangchote S. (2023). Polymer-Assisted Coprecipitation Synthesized Zinc Oxide Nanoparticles and Their Uses for Green Chemical Synthesis via Photocatalytic Glucose Conversions. ACS Omega.

[18] Mornani E.G., Mosayebian P., Dorranian D., Behzad K. (2016). Effect of calcination temperature on the size and optical properties of synthesized ZnO nanoparticles. J. Ovonic Res..

[19] Setiadji S., Aprilia V. (2025). Effect of calcination temperature and heating rate on zinc oxide synthesis toward antibacterial properties. J. Kim. Sains Apl..

[20] Singleton V.L., Orthofer R., Lamuela-Raventós R.M. (1999). Analysis of total phenols and other oxidation substrates and antioxidants by means of Folin-Ciocalteu reagent. Methods Enzymol..

[21] Chang C.C., Yang M.H., Wen H.M., Chern J.C. (2002). Estimation of total flavonoid content in propolis by two complementary colometric methods. J. Food Drug Anal..

[22] Brand-Williams W., Cuvelier M.E., Berset C. (1995). Use of a free radical method to evaluate antioxidant activity. LWT—Food Sci. Technol..

[23] Ceyhan O.S., Erkmen S. (2023). Identification of phenolic compounds and changes in their content during processes of white wines. Carpathian J. Food Sci. Technol..

[24] Fakhari S., Jamzad M., Kabiri Fard H. (2019). Green synthesis of zinc oxide nanoparticles: A comparison. Green Chem. Lett. Rev..

[25] Schofield C.B. (2012). Updating antimicrobial susceptibility testing methods. Clin. Lab. Sci. J..

[26] Emami-Karvani P.C. (2021). Antibacterial activity of ZnO nanoparticle on Gram-positive and Gram-negative bacteria. Afr. J. Microbiol. Res..

[27] Macwan D., Verma D., Patel H.V. (2022). Antioxidant capacities of crude extract and protein glycation inhibitory activities of bioactive fractions of *Arthrospira platensis* Gomont. Ann. Phytomed..

[28] Tong Liang S.X., Djearamane S., Tanislaus Antony Dhanapal A.C., Wong L.S. (2022). Impact of silver nanoparticles on the nutritional properties of *Arthrospira platensis*. PeerJ.

[29] Sari L.M., Rilda Y. (2023). Biosynthesis of ZnO nanoparticles using *Spirulina platensis* based on calcination temperature changes and its antioxidant activity. Chem. Sci. Int. J..

[30] Madunić J., Madunić I.V., Gajski G., Popić J., Garaj-Vrhovac V. (2018). Apigenin: A dietary flavonoid with diverse anticancer properties. Cancer Lett..

[31] Cock I.E., Cheesman M.J. (2023). A review of the antimicrobial properties of cyanobacterial natural products. Molecules.

[32] Alwash A. (2025). Impact of calcination temperature on the structural and photocatalytic properties of ZnO synthesized from gum arabic for methylene blue dye removal. J. Hazard. Mater. Adv..

[33] Dey S., Mohanty D.L., Divya N., Bakshi V., Mohanty A., Rath D., Das S., Mondal A., Roy S., Sabui R. (2025). A critical review on zinc oxide nanoparticles: Synthesis, properties and biomedical applications. Intell. Pharm..

[34] Sánchez-Pérez D.M., Flores-Loyola E., Márquez-Guerrero S.Y., Galindo-Guzman M., Marszalek J.E. (2023). Green Synthesis and Characterization of Zinc Oxide Nanoparticles Using *Larrea tridentata* Extract and Their Impact on the In-Vitro Germination and Seedling Growth of *Capsicum annuum*. Sustainability.

[35] Albarakaty F.M., Alzaban M.I., Alharbi N.K., Bagrwan F.S., Abd El-Aziz A.R.M., Mahmoud M.A. (2023). Zinc oxide nanoparticles, biosynthesis, characterization and their potent photocatalytic degradation, and antioxidant activities. J. King Saud Univ. Sci..

[36] Jayachandran A.T.R., Nair A.S. (2021). Green synthesis and characterization of zinc oxide nanoparticles using *Cayratia pedata* leaf extract. Biochem. Biophys. Rep..

[37] Giri P.K., Bhattacharyya S., Singh D.K., Kesavamoorthy R., Panigrahi B.K., Nair K.G.M. (2007). Correlation between microstructure and optical properties of ZnO nanoparticles synthesized by ball milling. J. Appl. Phys..

[38] Selim Y.A., Azb M.A., Ragab I., Abd El-Azim M.H.M. (2020). Green synthesis of zinc oxide nanoparticles using aqueous extract of *Deverra tortuosa* and their cytotoxic activities. Sci. Rep..

[39] Muhammad W., Ullah N., Haroon M., Abbasi B.H. (2019). Optical, morphological and biological analysis of zinc oxide nanoparticles (ZnO NPs) using *Papaver somniferum* L.. RSC Adv..

[40] Alallam B., Doolaanea A.A., Alfatama M., Lim V. (2023). Phytofabrication and characterisation of zinc oxide nanoparticles using pure curcumin. Pharmaceuticals.

[41] Dousti B., Habibi A., Nabipor F. (2021). Biosynthesis of zinc oxide nanoparticles using *Fumaria parviflora* extract and evaluation of their antibacterial and antioxidant activities. Biotechnologia.

[42] Jaishi D.R., Ojha I., Bhattarai G., Baraili R., Pathak I., Ojha D.R., Shrestha D.K., Sharma K.R. (2024). Plant-mediated synthesis of zinc oxide (ZnO) nanoparticles using *Alnus nepalensis* D. Don for biological applications. Heliyon.

[43] Farag M., El-Dafrawy S.M., Hassan S.M. (2024). ZnO and C/ZnO catalysts synthesized via plant mediated extracts for photodegradation of crystal violet and methyl orange dyes. J. Inorg. Organomet. Polym. Mater..

[44] Droepenu E.K., Amenyogbe E., Boatemaa M.A., Opoku E. (2024). Study of the antimicrobial activity of zinc oxide nanostructures mediated by two morphological structures of leaf extracts of *Eucalyptus radiata*. Heliyon.

[45] Jalal M., Abdelmohsen U.R., El-Helow A., El-Sheekh M.M. (2018). Anticandidal activity of bioinspired ZnO nanoparticles. J. Mycol. Fungal Biol..

[46] Raghupathi K.R., Koodali R.T., Manna A.C. (2011). Size-dependent bacterial growth inhibition and mechanism of antibacterial activity of zinc oxide nanoparticles. Langmuir.

[47] Krishnaveni P., Anjali P., Renuka S., Deivakumari M., Saranya V.P., Namasivayam S.K.R., Samrat K., Nachiyar C.V. (2025). Sustainable formulation of PEGylated zinc oxide nanoparticles to enhance plant growth and combat bacterial pathogens. Discov. Biotechnol..

[48] Kayani Z.N., Saleemi F., Batool I. (2015). Effect of calcination temperature on the properties of ZnO nanoparticles. Appl. Phys. A.

[49] Patil M.P., Kim J., Seo Y.B., Kang M., Kim G. (2021). Biogenic synthesis of metallic nanoparticles and their antibacterial applications. J. Life Sci..

[50] Elshafie H.S., Osman A., El-Saber M.M., Camele I., Abbas E. (2023). Antifungal activity of green and chemically synthesized ZnO nanoparticles against *Alternaria citri*, the causal agent citrus black rot. Plant Pathol. J..

[51] Çelekli A., Akhras N., Bozkurt H. (2024). Improving antimicrobial activity of brown propolis with incorporation of *Arthrospira platensis*. Food Humanit..

[52] Sharbatdaran M., Janbazi M. (2024). Effect of temperature on the structure, catalyst and magnetic properties of undoped zinc oxide nanoparticles: Experimental and DFT calculation. RSC Adv..

[53] Venu Gopal V.R., Kamila S. (2017). Effect of temperature on the morphology of ZnO nanoparticles: A comparative study. Appl. Nanosci..

